# Computed tomography findings in acute decompensated heart failure: periportal collar sign and lymphedema in the hepatoduodenal ligament and retroperitoneal space

**DOI:** 10.1186/s40064-015-1100-x

**Published:** 2015-06-21

**Authors:** Hiroyuki Takeda, Kenichi Kamachi, Toshiya Yoshida, Hirotaka Miyazono, Tadao Kuruma, Shigeru Fujii

**Affiliations:** Department of Radiology, Fukuoka Shin Mizumaki Hospital, 1-2-1 Tateyashiki, Mizumaki-machi, Onga-gun, Fukuoka, 807-0051 Japan; Department of Surgery, Fukuoka Shin Mizumaki Hospital, 1-2-1 Tateyashiki, Mizumaki-machi, Onga-gun, Fukuoka, 807-0051 Japan; Department of Cardiovascular Medicine, Fukuoka Shin Mizumaki Hospital, 1-2-1 Tateyashiki, Mizumaki-machi, Onga-gun, Fukuoka, 807-0051 Japan

**Keywords:** Periportal collar sign, Lymphedema, Retroperitoneal space, Acute decompensated heart failure, CT finding

## Abstract

We report two cases that were diagnosed with either acute exacerbation of chronic heart failure or acute decompensated heart failure due to acute myocardial infarction; both cases exhibited computed tomography (CT) findings of the periportal collar sign in the liver and lymphedema in the hepatoduodenal ligament and retroperitoneal space. Both of these signs, particularly lymphedema in the hepatoduodenal ligament and anterior pararenal space, are considered very important CT findings when diagnosing acute decompensated heart failure.

## Background

Periportal collar sign, which is characterized by areas of low attenuation around the portal vein and its branches, is a well-known computed tomography (CT) finding in patients with congestive heart failure or other conditions such as hepatic transplantation (Lang et al. 1995; Stevens et al. 1991), hepatic trauma, acute hepatitis, cholangitis, malignant neoplasm of the liver and bile duct, masses, enlarged lymph nodes in the porta hepatis, and extramedullary hematopoiesis (Koslin et al. 1988; Matsui et al. 1989; Lawson et al. 1993). However, lymphedema in the hepatoduodenal ligament and retroperitoneal space is not a well-known CT finding in patients with acute decompensated heart failure. We report here two cases of acute decompensated heart failure that presented with CT findings of not only the periportal collar sign, but also lymphedema in the hepatoduodenal ligament and retroperitoneal space with minimal ascites.

## Case reports

### Case 1

An 83-year-old woman with the chief complaint of reduced consciousness and slight chest pain was transferred to our hospital by ambulance.

Upon arrival, her face and eyelid conjunctiva were pale and her distal limbs were cold. Her clinical values were as follows: level of consciousness, Japan Coma Scale (JCS)-1; blood pressure, 99/36 mmHg; heart rate, 128/min; body temperature, 35.8°C; and oxygen saturation (SpO_2_), 98% in room air. Her blood laboratory data revealed inflammation, with a C-reactive protein (CRP) level of 12.84 mg/dL and white blood cell count of 16,100/µL (neutrophils, 89.9%), and anemia, with a hemoglobin concentration of 5.8 g/dL and hematocrit of 19.6%. Her hepatic, biliary, pancreatic, and renal functions were almost normal. However, the titers of creatine phosphokinase (CPK; 403 IU/L) and brain natriuretic peptide (BNP; 738.3 pg/ml) were elevated. The patient had a previous history of hypothyroidism, but her thyroid function had normalized with medication.

**Figure 1:**
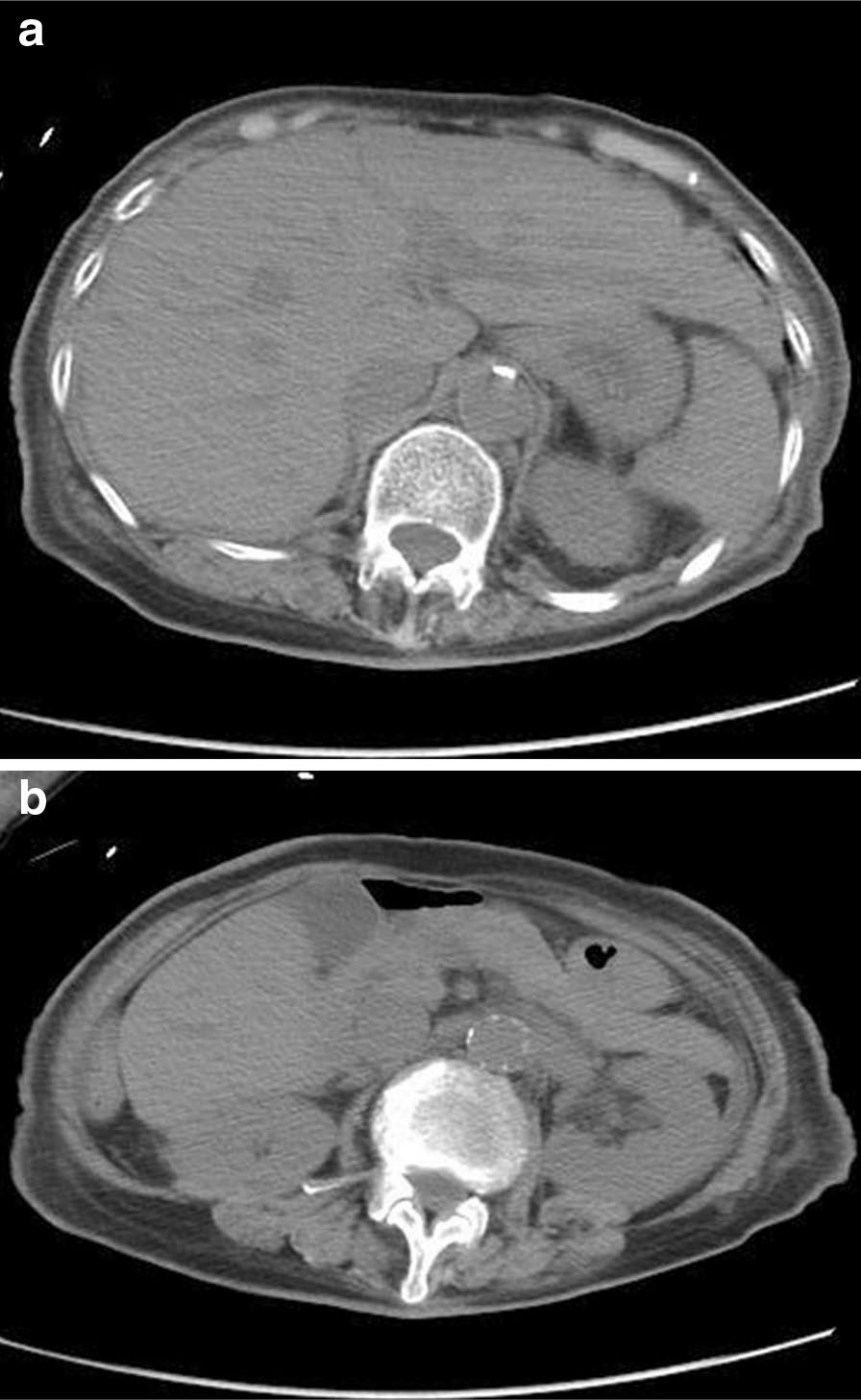
Axial non-contrast computed tomography at the level of the left portal vein of the liver (**a**) and pancreatic body (**b**). No abnormal findings were observed.

Non-contrast CT performed soon after arrival revealed no abnormality (Figure 1).

**Figure 2:**
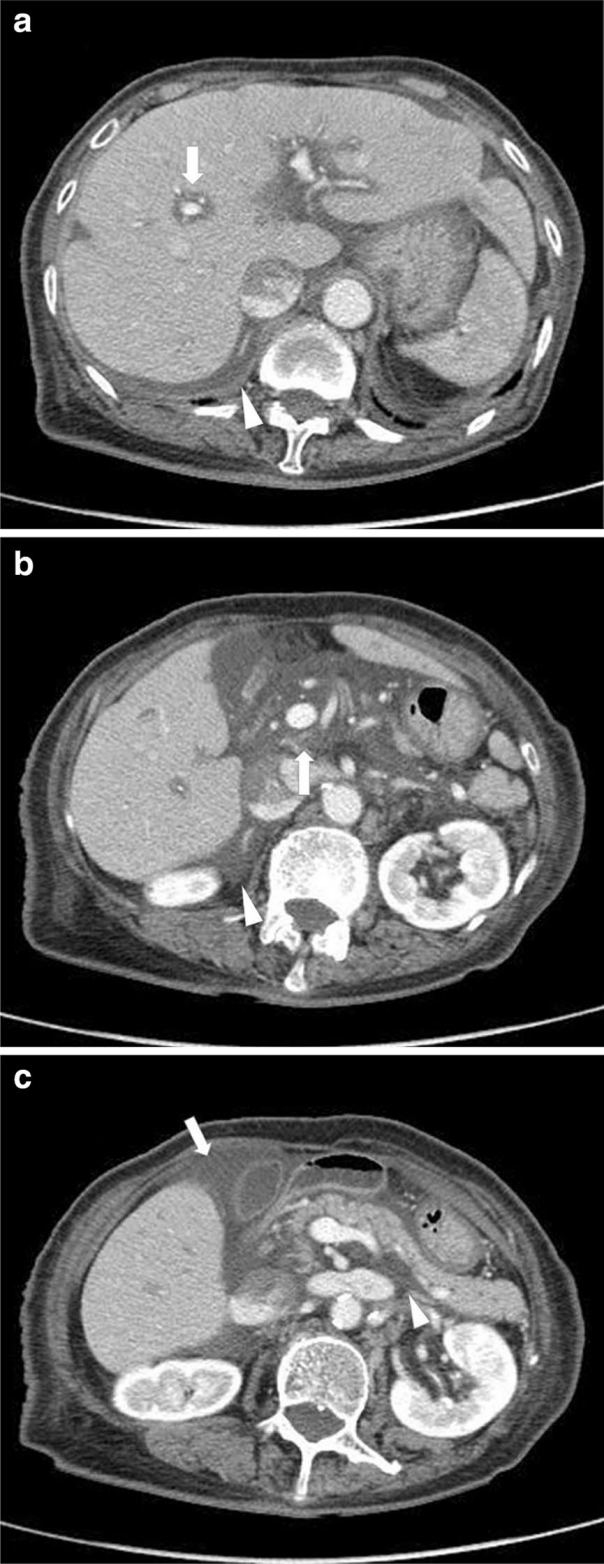
Axial contrast-enhanced computed tomography at the level of left portal vein of the liver (**a**), main portal vein (**b**), and pancreatic body (**c**). The periportal collar sign (*arrow*) and lymphedema in the bare area of the liver (*arrowhead*) are depicted in (**a**). Lymphedema in the hepatoduodenal ligament (*arrow*) and bare area of the liver (*arrowhead*) are depicted in (**b**). Subserosal edema of the gallbladder wall (*arrow*) and lymphedema in the anterior pararenal space (*arrowhead*) are depicted in (**c**).

Approximately 4 h after the initial non-contrast CT, contrast-enhanced CT was performed without non-contrast CT. This examination revealed areas of low density surrounding the portal veins in the liver (periportal collar sign), subserosal edema of the gallbladder wall, and areas of water density in the hepatoduodenal ligament, anterior pararenal space, and bare area of the liver with minimal ascites in the right subphrenic space (Figure 2).

Echocardiography was performed for suspected acute congestive heart failure. This evaluation revealed diffuse hypokinesis of the left ventricular wall motion, decreased ejection fraction (by Simpson method) of 45.34%, moderate aortic regurgitation, mitral regurgitation and tricuspid regurgitation. The right ventricular systolic pressure was estimated at approximately 40 mmHg. Acute exacerbation in chronic heart failure was diagnosed in accordance with the above-mentioned cardiac function findings.

### Case 2

A 90-year-old man with the chief complaint of respiratory distress was transferred by ambulance. He had a past history of diabetes mellitus, hypertension, and urinary bladder cancer. Upon arrival, his consciousness was clear, blood pressure was 126/69 mmHg, heart rate was 96/min, body temperature was 36.1°C, and SpO_2_ was 100% under mask ventilation with an oxygen flow rate of 10 L/min. Blood laboratory data revealed moderate liver and kidney dysfunction and inflammation.

**Figure 3:**
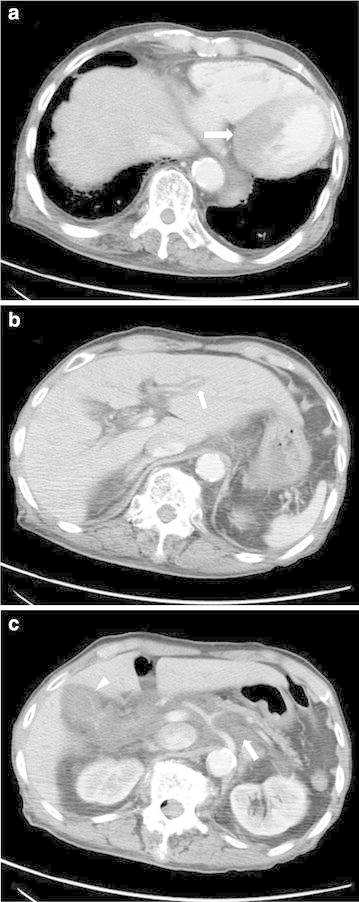
Axial contrast-enhanced computed tomography at the level of the heart (**a**), left portal vein of the liver (**b**), and pancreatic body (c). Part of the left ventricular wall (*arrow*) is not enhanced in (**a**). The periportal collar sign (*arrow*) is depicted in (**b**). Lymphedema in the anterior pararenal space (*arrow*) and subserosal edema of the gallbladder wall (*arrowhead*) are depicted in (**c**).

Contrast-enhanced CT revealed areas of low density surrounding the portal veins in the liver (periportal collar sign), subserosal edema of the gallbladder wall, areas of water density in the hepatoduodenal ligament and the anterior pararenal space, and minimal ascites in the Douglas pouch. Acute myocardial infarction was suspected because part of the left ventricular wall was not enhanced (Figure 3). Echocardiography revealed diffuse hypokinesis of the left ventricular wall motion, decreased ejection fraction (by Simpson method) of 27%, mild mitral regurgitation and tricuspid regurgitation. The right ventricular systolic pressure was estimated to be approximately 34 mmHg. Coronary angiography, which was performed immediately, indicated right coronary artery occlusion and severe left circumflex artery stenosis. The patient was diagnosed with acute decompensated heart failure due to acute myocardial infarction.

## Discussion

Hepatic lymph primarily derives from the hepatic sinusoids. Fluid filters out of the sinusoids and into the space of Disse to finally enter the lymphatic vessels in Glisson’s sheath (connective tissue containing the portal vein, hepatic artery, biliary duct, and lymphatic vessels) and become lymph. The hepatic drainage volume in a normal adult is approximately 1–3 L/day. An estimated 25–50% of the lymph received by the thoracic duct originates in the liver. The hepatic lymphatics are divided into deep system lymphatics (those following the hepatic veins and portal tract) and superficial system lymphatics (convex and inferior surfaces) (Pupulim et al. 2015; Ohtani and Ohtani 2008). Under normal conditions, lymph production and discharge are in equilibrium; as a result, no lymphatic dilatation or lymph retention is present and thus cannot be depicted on CT. However, under abnormal conditions such as lymph overproduction and dysregulated drainage, CT can depict lymphatic dilatation and lymph retention as areas of low density in the periportal spaces. For example, because metastases to hepatic hilar lymph nodes or direct malignant invasion of the hepatic hilum disturb lymph drainage from the liver, hepatic lymphatic dilatation and lymph retention in the periportal space are depicted as areas of low density on CT (periportal collar sign).

In acute decompensated heart failure (biventricular failure), an acute increase in venous pressure disrupts the lymphatic flow from the liver that drains into the left subclavian vein via a pressure gradient. Increased venous pressure in the hepatic veins and sinusoids is thought to cause hepatic lymph overproduction. Hepatic lymphatic flow disruption and hepatic lymph overproduction are thought to cause lymphatic vessel dilatations and lymph retention in Glisson’s sheath, which is depicted on CT as areas of low density (periportal collar sign). Lymph that is not absorbed by the lymphatic vessels is thought to overflow into potential subperitoneal spaces created by the hepatic ligaments (e.g., hepatoduodenal and gastrohepatic ligaments), as well as retroperitoneal spaces (e.g., anterior pararenal space and bare area of the liver). In our cases, these phenomena were thought to have been depicted on CT as areas of water density (i.e., lymphedema) in the hepatoduodenal ligament, gastrohepatic ligament (which cannot be distinguished from ascites in the lesser sac), anterior pararenal space, and bare area of the liver.

Koyama et al. reported a case of traumatic cardiac rupture with acute ascites (Koyama et al. 2000). In that article, an emergency sternotomy revealed a rupture of the right atrial appendage. Laparotomy was performed to aspirate a moderate volume of clear, non-bloody serous fluid. Retroperitoneal space edema and hepatic congestion were noted without accompanying organ injury. Abdominal CT with contrast medium demonstrated irregular staining in the liver, dilatation of the portal vein associated with a periportal low-density band and gallbladder wall thickening, and moderate peritoneal effusion in the pelvic cavity; however, there have been no reports of edema of the hepatoduodenal ligament and retroperitoneal space.

The periportal collar sign is a well-known CT finding associated with congestive heart failure; however, lymphedema in the hepatoduodenal and gastrohepatic ligaments, retroperitoneal space (e.g., anterior pararenal space and bare area of the liver) is not a well-known CT finding associated with this disease, particularly in cases of acute decompensated heart failure.

The periportal collar sign is most easily detected in the portal venous phase on contrast-enhanced CT (Kanazawa et al. 1999) and most difficult to detect with on non-contrast CT; however, lymphedema in the hepatoduodenal ligament and retroperitoneal space is more easily detected even on non-contrast CT. On non-contrast CT only, the observance of lymphedema in the hepatoduodenal ligament and retroperitoneal space in addition to the periportal collar sign provides an important clue leading to the suspicion of acute decompensated heart failure. From this viewpoint, we insist that lymphedema in the hepatoduodenal ligament and retroperitoneal space, particularly anterior pararenal space, is a very important CT finding.

Lymphedema in the hepatoduodenal ligament and retroperitoneal space were observed in some cases that exhibited periportal collar sign on CT as well as in some cases of acute decompensated heart failure. However, the frequency and etiology of lymphedema in the hepatoduodenal ligament and retroperitoneal space are unknown. We speculate that the etiology of lymphedema correlates with the severity of acute decompensated heart failure (biventricular failure), degrees of venous pressure and circulating blood volume, presence of dehydration or overhydration, and time interval between the onset of acute decompensated heart failure and CT examination, among other factors.

## Conclusion

Herein, we reported two cases with diagnosis of acute decompensated heart failure; both cases exhibited the periportal collar sign in the liver on CT as well as lymphedema in the hepatoduodenal ligament and retroperitoneal space. Both of these CT findings, particularly lymphedema in the hepatoduodenal ligament and anterior pararenal space, are very important when diagnosing acute decompensated heart failure.

The frequency and etiology of CT-detected lymphedema in the hepatoduodenal ligament and retroperitoneal space are unknown among cases of acute decompensated heart failure; therefore, further investigation and analysis of CT findings, together with clinical findings and laboratory data, in sequential cases are needed to elucidate this information.

### Consent

Written informed consent for the publication of this report and any accompanying images was obtained from all patients.

## Abbreviations

CT: Computed tomography
JCS: Japan coma scale
SpO_2_: oxygen saturation
CRP: C-reactive protein
CPK: creatine phosphokinase
BNP: brain natriuretic peptide

## References

[CR1] Kanazawa S, Tanaka A, Yasui K, Akaki S, Hiraki Y (1999). Attenuation changes in periportal region during triple-phasic contrast-enhanced CT. Radiat Med.

[CR2] Koslin DB, Stanley RJ, Berland LL, Shin MS, Dalton SC (1988). Hepatic perivascular lymphedema: CT appearance. AJR Am J Roentgenol.

[CR3] Koyama T, Miyamoto S, Murakami H, Kitanaka Y, Ikeshita M, Yamate N (2000). Traumatic cardiac rupture with acute ascites. Jpn J Thorac Cardiovasc Surg.

[CR4] Lang P, Schnarkowski P, Grampp S, van Dijke C, Gindele A, Steffen R (1995). Liver transplantation: significance of the periportal collar on MRI. J Comput Assist Tomogr.

[CR5] Lawson TL, Thorsen MK, Erickson SJ, Perret RS, Quiroz FA, Foley WD (1993). Periportal halo: a CT sign of liver disease. Abdom Imaging.

[CR6] Matsui O, Kadoya M, Takashima T, Kameyama T, Yoshikawa J, Tamura S (1989). Intrahepatic periportal abnormal intensity on MR images: an indication of various hepatobiliary diseases. Radiology.

[CR7] Ohtani O, Ohtani Y (2008). Lymph circulation in the liver. Anat Rec (Hoboken).

[CR8] Pupulim LF, Vilgrain V, Ronot M, Becker CD, Breguet R, Terraz S (2015) Hepatic lymphatics: anatomy and related diseases. Abdom Imaging [Epub ahead of print] 10.1007/s00261-015-0350-y 25579171

[CR9] Stevens SD, Heiken JP, Brunt E, Hanto DW, Flye MW (1991). Low-attenuation periportal collar in transplanted liver is not reliable CT evidence of acute allograft rejection. AJR Am J Roentgenol.

